# Dynamic Multiscale Information Spillover among Crude Oil Time Series

**DOI:** 10.3390/e24091248

**Published:** 2022-09-05

**Authors:** Sufang An

**Affiliations:** 1 College of Information and Engineering, Hebei GEO University, Shijiazhuang 050031, China; fishwater3927@gmail.com or bjxsky@126.com; 2 School of Economics and Management, China University of Geosciences, Beijing 100083, China; 3 Intelligent Sensor Network Engineering Research Center of Hebei Province, Shijiazhuang 050031, China

**Keywords:** spillover network, wavelet transform, oil time series, system risk entropy

## Abstract

This study investigated information spillovers across crude oil time series at different time scales, using a network combined with a wavelet transform. It can detect the oil price, which plays an important role in the dynamic process of spillovers, and it can also analyze the dynamic feature of systematic risk based on entropy at different scales. The results indicate that the network structure changes with time, and the important roles of an oil price can be identified. WTI and Brent act as important spillover transmitters, and other prices are important spillover receivers at a scale. With the increase in time scale, both the number of neighbors and the importance of spillovers of Brent and WTI as spillover transmitters show downward trends. The importance for spillovers of China–Shengli and Dubai as spillover receivers shows a downward trend. This paper provides new evidence for explaining WTI and Brent as global benchmark oil prices. In addition, systematic risk is time-varying, and it is smaller at short-term scale than at long-term scale. The trend of systematic risk is also discussed when typical oil-related events occur. This paper provides a new perspective for exploring dynamic spillovers and systematic risk that offers important implications for policymakers and market investors.

## 1. Introduction

Crude oil is a crucial strategic resource and is one of the important commodities in global financial markets [1,2]. The fluctuation of the oil price is the key input into the macroeconomic and financial risk models [3], which has been influenced by oil-related events, such as the global financial crisis of 2007–2009 and the COVID-19 pandemic [4]. In recent years, most researchers support the integration of the world oil market [5,6]. This indicates that an event in one market can quickly spread to the other oil markets. This has attracted much attention from researchers to investigate information spillovers across oil time series, which can help to understand the risk contagion in risk management [7]. There are a large number of spillover models, such as the FIAPARCH–DCC model [8], structural VAR model and copula-GARCH model [9], VAR–BEKK–AGARCH model [10], volatility spillover decomposition method [11], and a dynamic network based on the GARCH–BEKK model [6]. These models range from a static perspective to a dynamic perspective to investigate spillovers. However, stakeholders pay attention to different time scales of oil price fluctuations [12]. For instance, policymakers focus on the long-term features of price trends, while market investors are concerned about short-term prices to gain profits. Thus, there is a need for research on information spillovers across oil series from different time scales.

The wavelet transform is a famous mathematical tool that can decompose a time series into several time scales through wavelet functions. The method has been widely applied in financial markets. For instance, Yu et al. [13] examined the causality between the carbon market and crude oil market by using bivariate empirical mode decomposition (BEWD). Huang, An, Wen, and An [12] established the vector autoregression (VAR) model and a torus wavelet transform model to study the driving factors of oil price shocks at different time scales. Conlon et al. [14] measured the relationship between cryptocurrencies and forward inflation expectations on different time scales, using a wavelet time-scale model. Kuang [15] proposed an entropy-based network to investigate information flows across stock markets on multiple scales, where they used empirical mode decomposition (EMD) to reconstruct the stock index series. Wen et al. [16] built a MODWT-Vine quantile regression method to investigate the risk contagion effect among the international oil market and the commodity and stock markets in China under multiple scales. Mastroeni et al. [17] exploited wavelet analysis and energy-based measures to explore the oil–food price relationship in the time–frequency domains. Previous studies focused on financial time series at different scales, where the main feature of these models is the wavelet transform model combined with the economic model. However, they rarely considered the dynamic structure of the spillover relationships across financial time series at different time scales. It can help us understand the hidden information of time series in the dynamic process and can provide evidence for different stakeholders.

The aim of this paper is to investigate the information spillovers across multivariate oil price time series at different time scales. The China–Daqing and China–Shengli crude oil spot prices and their four reference benchmark oil prices are selected as the sample data. China is the largest importer of crude oil in the world. Hence, the crude oil price fluctuation in China is representative. The main contributions of this paper are twofold. This paper first establishes a network model based on the spillover model and a wavelet transform. The original time series is decomposed into several time series at different scales. At each time scale, the dynamic network connectedness model is built to investigate the dynamic process of information spillovers across oil prices. Second, the important role of an oil price in an information spillover network can be detected at different time scales. The systematic risk of the oil system consisting of oil prices can be analyzed based on entropy theory and the structure of the network as a whole.

The structure of this paper is as follows. Section 2 presents the materials and methods. Section 3 shows the results. Finally, conclusions and discussions are drawn in Section 4.

## 2. Materials and Methods

### 2.1. Materials

This paper selects the China–Daqing and China–Shengli crude oil spot prices and their four reference benchmark oil prices as the sample data. The WTI and Brent crude oil future prices are the global representative benchmark oil prices. Dubai and Minas crude oil spot prices can be selected according to the China pricing rules. This paper covers the daily crude oil price in each time series, from 13 July 2012, to 10 March 2022. Each time series consists of 2333 daily crude oil prices (US dollars per barrel), and the database can be obtained from the wind database and the websites of the Energy Information Administration (http://www.eia.gov).

**Figure 1:**
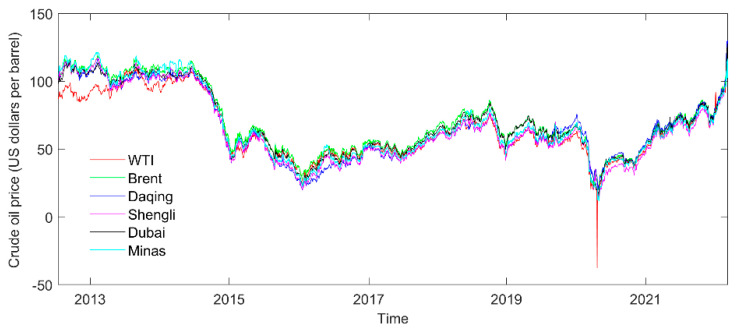
The original database of crude oil prices.

The distribution characteristics are described in Table 1. Brent has the greatest maximum crude oil prices, Dubai has the highest mean, and Daqing has the greatest standard deviation. Original sample data of the crude oil prices are shown in Figure 1. Daily data over a long period are selected since information transmission across oil markets is fast and fluctuations in oil prices are affected by oil-related events, such as the COVID-19 pandemic. In addition, WTI crude oil prices have a negative value. This paper uses the minimum value of the history of crude oil prices instead of it.

### 2.2. Methods

In this section, the two main methods are introduced. The first is the decomposition of the oil time series into sub time series with different time scales based on the wavelet transform model. The second is the construction of the dynamic information spillover network combined with the rolling-window method and the network-connectedness model in each time scale. Then the dynamic topological structure of each node, the topological structure of a network as a whole, and the system risk entropy are measured to examine the dynamic information spillovers among different oil time series.

#### 2.2.1. The Dynamic Information Spillover Network

**Step 1**. Obtaining oil series at multiple scales based on the MODWT method:

Wavelet transforms are an efficient tool to analyze time series at multiple scales, and they have a good effect on nonstationary series analysis since they combine information from both time and frequency [18]. It can help to capture the volatile behavior of time series, such as breakpoints and trends [19]. A financial time series in the real world is inherently nonstationary and usually has seasonal variations and long-term and short-term fluctuations. In recent years, some researchers and scholars have paid much attention to hybrid models combined with economic models and wavelet models [12,13,18] to investigate the relationship among different financial time series at multiple time scales. The main idea of this framework is to transform the time series into several series at different time scales based on a wavelet model and then to establish economic models to reveal the relationship among time series at a time scale. The maximal overlap discrete wavelet transformation method (MODWT), which is widely applied in economics, can be used to map the whole oil time series into sub time series at short-, medium-, and long-term time scales [20,21].

In a discrete wavelet transformation method (DWT), l (l=1,2,⋯,L) represents the filter length, j (j=1,2,⋯,J) represents the decomposition scale, and DWT consists of wavelet filter $\left\{d_{j,l}\right\}$ and scaling filter $\left\{s_{j,l}\right\}.$ In MODWT, wavelet $\left\{{\tilde{d}}_{j,l}\right\}$ and scaling filters $\left\{{\tilde{s}}_{j,l}\right\}$ can be defined as ${\tilde{d}}_{j,l}=d_{j,l}/2^{j/2}$ and ${\tilde{s}}_{j,l}=s_{j,l}/2^{j/2}$. Thus, the wavelet and scale coefficients for the original time series, ${\left\{X_{t}\right\}}_{t=0}^{N-1}$, can be denoted as follows:

(1) $${\tilde{W}}_{j,t}=\sum_{l=0}^{\;L_{j}-1}{\tilde{d}}_{j,l}X_{t-l\mathrm{mod}N}$$

(2) $${\tilde{S}}_{j,t}=\sum_{l=0}^{\;L_{j}-1}{\tilde{s}}_{j,l}X_{t-l\mathrm{mod}N}$$

where $L_{j}$ = ($2^{j}-1$) (L−1) + 1. According to the definition of the MODWT, the wavelet coefficients at a scale have the same length as the original time series. Suppose ${\tilde{W}}_{j}={\tilde{\omega }}_{j}X$ and ${\tilde{S}}_{j}={\tilde{s}}_{j}X$, where both ${\tilde{\omega }}_{j}$ and ${\tilde{S}}_{j}$ are N×N matrices, and the elements of ${\tilde{\omega }}_{j}$ and ${\tilde{S}}_{j}$ are related to ${\tilde{d}}_{j,l}$ and ${\tilde{s}}_{j,l}$. Thus, the original time series can be reconstructed as follows:

(3) $$X=\sum_{j=1}^{J}{\tilde{\omega }}_{j}^{T}{\tilde{W}}_{j}+s_{j}^{T}{\tilde{S}}_{j}=\sum_{j=1}^{J}{\tilde{D}}_{j}+{\tilde{V}}_{j}$$

where ${\tilde{D}}_{j}={\tilde{\omega }}_{j}^{T}{\tilde{W}}_{j}$ represents the MODWT detail on the decomposition scale, j; and ${\tilde{V}}_{j}=s_{j}^{T}{\tilde{S}}_{j}$ is the trend level.

An oil time series, ${\left\{X_{t}\right\}}_{t=0}^{N-1}$, exists, and $X_{t}$ represents the oil price at time, t. If j (j=1,2,⋯,J) represents the decomposition scale, the sub time series, $\left\{P_{t,j}\right\},t=0,1,\cdots ,N-1$, is obtained from the oil series based on MODWT, where $P_{t,j}$ represents the value of a decomposed series at time (t) in scale j. If there are M oil time series, several sub time series will be obtained, and then these series at each scale can be reconstructed into a dynamic information spillover network.

**Step 2**. Reconstruct the dynamic information spillover network on a scale:

This study establishes a dynamic information spillover network based on the Diebold and Yilmaz model (DY model) [22,23,24]. First, a multivariable oil time series in a scale can be divided into several segments from left to right, using a rolling window [25], which can be denoted as $WIN={\left\{WIN_{w}\right\}}_{w=1}^{W}$. Note that the input data of the economic model should be stationary to support its assumption; if the decomposed time series is not stationary, the differential series can be used. In each segment, a DY model is used, which is combined with a vector autoregressive (VAR) model. The n-variable, VAR (p), model can be constructed as follows:

(4) $$x_{t}=\sum_{i=1}^{p}\varphi_{i}x_{t-i}+\varepsilon_{t}$$

where $x_{t}$ is an n×1 dimensional variable, $\varphi_{i}$ is an n×n autoregressive coefficient matrix, and $\varepsilon_{t}$ is the vector of disturbances, which is independently and identically distributed. The moving representation is $x_{t}$ = $\sum_{i=1}^{\infty }B_{i}\varepsilon_{t-i}$, where $B_{i}$ is an n×n coefficient matrix and can be described as $B_{i}=\varphi_{1}\;B_{i-1}+\varphi_{2}\;B_{i-2}+\cdots +\varphi_{p}\;B_{i-p}$. Note that $B_{0}$ is an n×n identity matrix, and $B_{i}=0$ for i<0.

The net spillovers among oil series at a scale are based on H-period-ahead forecast error variance decomposition, which can be denoted as follows:

(5) $$\theta_{ij}\left(H\right)=\frac{\theta_{ii}^{-1}\sum_{h=0}^{H-1}{\left(e_{i}^{\prime }B_{h}\sum e_{j}\right)}^{2}}{\sum_{h=0}^{H-1}\left(e_{i}^{\prime }B_{h}\sum B_{h}^{\prime }e_{i}\right)}$$

where Σ represents the variance matrix of the vector of error ε; $\theta_{ii}$ represents the standard deviation of the error term for the ith equation; and $e_{i}$ is the selector vector, where the ith element is one and zero otherwise.

The normalized variance decomposition matrix can be generated as follows:

(6) $${\tilde{\theta }}_{ij}\left(H\right)=\theta_{ij}\left(H\right)/\sum_{j=1}^{n}\theta_{ij}\left(H\right)$$

The directional spillover received by an oil series, i, from all other series, j, can be measured as follows:

(7) $$S_{i\leftarrow j}\left(H\right)=\frac{\sum_{j=1,j\neq i}^{n}{\tilde{\theta }}_{ij}\left(H\right)}{\sum_{i,j=1}^{n}{\tilde{\theta }}_{ij}\left(H\right)}\times 100$$

In a similar fashion, the information spillover received by an oil series, j, from all other series, i, at a time scale can be denoted as follows:

(8) $$S_{i\to j}\left(H\right)=\frac{\sum_{j=1,j\neq i}^{n}{\tilde{\theta }}_{ji}\left(H\right)}{\sum_{i,j=1}^{n}{\tilde{\theta }}_{ji}\left(H\right)}\times 100$$

Then the net information spillovers from each series to all other series at a time scale are as follows:

(9) $$IS_{i}=S_{i\to j}\left(H\right)-S_{i\leftarrow j}\left(H\right)$$

The net information spillover matrix for all series in a scale is mapped into an information spillover network based on network theory [26,27,28,29], where the node is the oil series and the weighted directed edge is the net information spillover from one series to the other.

**Figure 2:**
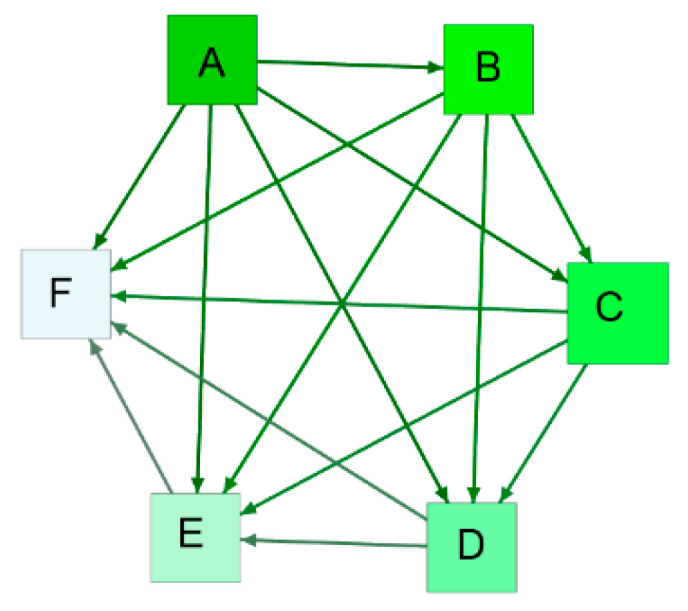
An example of an information spillover network.

Based on this algorithm, an information spillover network in a segment is obtained. Thus, a sequence of information spillover networks from the multivariate oil time series in a scale can be obtained, and these networks form an evolutionary process, which can be described as $Net={\left\{Net_{w}\right\}}_{w=1}^{W}$, where W is the number of information spillover networks on a time scale. Figure 2 shows an example of an information spillover network and indicates that there is a directed edge between any two series. For instance, the directed edge from A to D indicates that A acts as an information spillover transmitter and D acts as an information spillover receiver. The topological structures of these networks can reveal the dynamics of spillovers among oil series.

#### 2.2.2. Measures of Spillovers across Oil Prices

This paper analyzes the out degree, out strength, and in strength of a single node at each scale to detect the important role of an oil series in an information spillover network on a scale. Systematic risk is an important indicator in risk management [30,31,32,33]. This paper uses the total spillover and the system risk entropy to describe the dynamic systematic risk of the system consisting of oil series at a time scale.

1. Measure for a single oil series

**Out degree**:

The out degree of a node is a basic indicator of a network that describes the number of links from the node to its neighbors. The out degree of a node in an information spillover network calculates the number of information spillovers that flow from the node to its neighbors. The out degree of node i in an information spillover network, $Net_{w}$, is defined as follows:

(10) $$OD{\left(i\right)}_{w}=\sum_{j\in Q}a_{ij}^{w}$$

where Q represents the set of the node’s neighbors in the network, $Net_{w}$; $a_{ij}^{w}$ is the link from node i to node j; and $a_{ij}^{w}=1$ indicates that a link exists, while $a_{ij}^{w}=0$ reflects that there exists no link. A greater out degree of a node suggests that there are many information-spillover flows from the node to its neighbors. Note that any two nodes have a directed edge connecting them. If there are more information spillover flows from a node to its neighbors, there are fewer spillover flows received by this node.

**Out strength**:

The out strength of a node is an important indicator of a network that measures the numbers and weights from the node to its neighbors. This suggests the importance of the information spillover sent by the oil series to its neighbors. The out strength of node i in an information spillover network, $Net_{w}$, is denoted as follows [34]:

(11) $$OS{\left(i\right)}_{w}=\sum_{j\in Q}w_{ij}^{w}$$

where Q represents the set of the node’s neighbors in network, $Net_{w}$; and $w_{ij}^{w}$ is the weight from node i to node j. The higher indicator reflects the higher importance of information spillover sent by the oil series to other oil series.

**In strength**:

The strength of a node is a basic indicator of a network that measures the numbers and weights received by the node from its neighbors. This suggests the importance of the information spillover received by the oil series from its neighbors. The strength of node i in an information spillover network, $Net_{w}$, is denoted as follows [34]:

(12) $$IS{\left(i\right)}_{w}=\sum_{j\in Q}w_{ji}^{w}$$

where Q represents the set of the node’s neighbors in the network, $Net_{w}$, and $w_{ji}^{w}$ is the weight from node j to node i. The greater this indicator is, the greater the importance of information spillover received by the oil series from other oil series.

2. Measure for an oil system

**System risk entropy**:

System risk entropy is a measure of systematic risk (or market risk) based on entropy theory [35]. This suggests the systematic risk of the oil system consisting of oil prices. Suppose the eigenvalues, $\lambda_{i},i=1,2,\ldots ,H$, of the correlation matrix in a segment, $WIN_{w}$, correspond to an information spillover network, $Net_{w}$. The elements of the matrix are related to Pearson’s correlation coefficient. M is the dimension of the correlation matrix, and H≤M. The system risk entropy can be defined as follows:

(13) $$SE=\left(-1/\log\left(M\right)\right)\;\sum_{i=1}^{H}\left(\;\lambda_{i}/M\right)\log\left(\lambda_{i}/M\right)$$

Entropy is a measure of confusion and disorder. A higher entropy of a system indicates a greater level of confusion. A greater system risk entropy suggests that the systematic risk is lower.

**Total spillover index**:

The total spillover is an indicator used to measure the feature of oil series integration. Based on Equation (6), it can be denoted as follows:

(14) $$TC=\frac{\sum_{i,j=1,j\neq i}^{n}{\tilde{\theta }}_{ij}\left(H\right)}{\sum_{i,j=1}^{n}{\tilde{\theta }}_{ij}\left(H\right)}\times 100$$

The degree of integration among oil prices indicates the systematic risk in this paper. A greater degree of integration indicates that the systematic risk is larger.

## 3. Results

Based on the proposed method, each oil time series is first reconstructed into several sub time series through a wavelet model, where the length of the sub time series is the length of the original time series. The time scale is set to six since it is appropriate for describing the fluctuation features of the financial time series [36]. Table 2 illustrates the details of the scales of the time series. In multiresolution theory, the similarity between the decomposed time series and the original time series may increase with increasing time scale. This indicates that the greatest similarity exists between the original time series and the decomposed time series at time scale D6. To ensure that all the VAR components are stationary, the augmented Dicker–Fuller (ADF), at the 1% significance level, can be used [37]. The results show that the sub oil time series from scale D1 to scale D5 are stationary, and the differential data of the time series at scale D6 can be used since it is stationary. Second, a rolling window is used to divide the whole series at the same scale into thousands of segments. The window width is an economic cycle of 240 days, which is equal to one year when weekends and holidays are deleted. Third, the information spillover matrix in each segment is calculated, and then an information spillover network can be obtained. Fourth, the topological structure and system risk entropy in each network can be measured. Then the role of each oil price can be detected, and the systematic risk can be analyzed at the short-, medium-, and long-term time scales.

### 3.1. Dynamic Structure of an Oil Series

**Figure 3:**
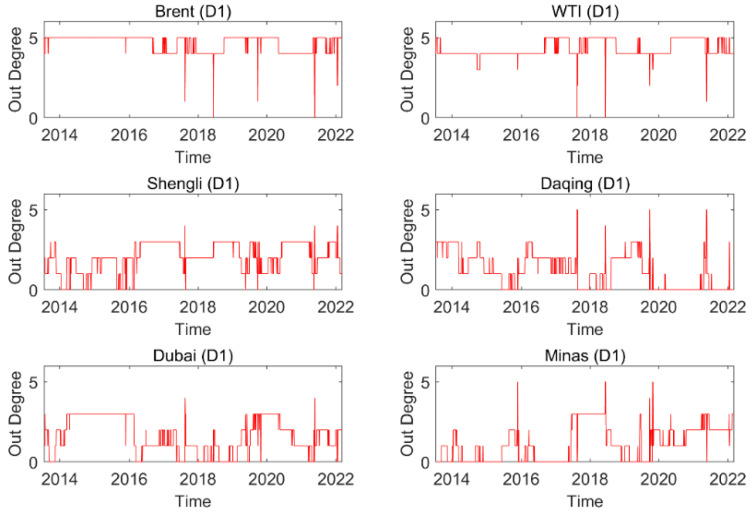
Dynamic out degree of each oil series on time scale D1.

**Figure 4:**
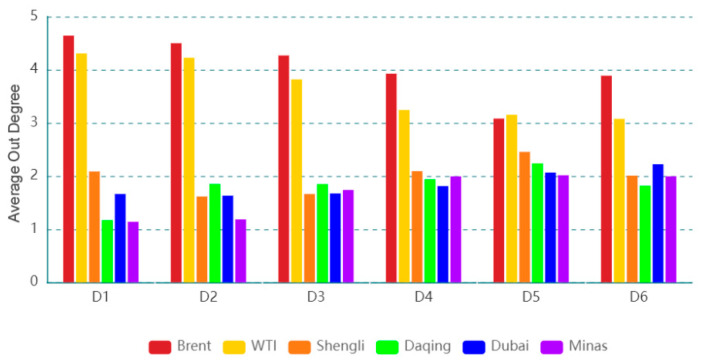
Average out degree of each oil series on different time scales.

Figure 3 exhibits the dynamic out degree for each oil series on a time scale (D1). The dynamic out degree of each oil series on the other time scales is shown in the Appendix A. The results imply that the number of information spillover flows from an oil series and its neighbors in the time scale is time-varying. The dynamic features in different time scales of out degree are different, indicating that the dynamic feature is related to time scales. Furthermore, the average out degree of an oil series at different time scales is given in Figure 4. For instance, on a time scale D1, Brent has the highest average out degree, and Minas has the smallest value. This reflects that Brent, as an information spillover transmitter, has the largest number of neighbors, and Minas has the lowest number of neighbors. In addition, with the increase in the time scale, the average out degree of Brent (WTI) shows a downward trend, and that of Daqing (Dubai/Minas) exhibits an upward trend. This indicates that the number of neighbors of Brent (WTI), as a spillover transmitter, shows a download trend and that of Daqing (Dubai/Minas), as a spillover transmitter, shows an upward trend with the increase in the time scales.

**Figure 5:**
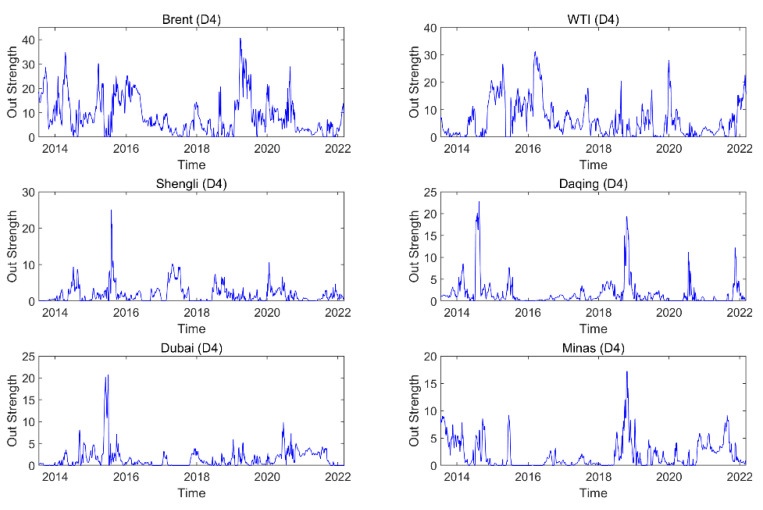
Dynamic out strength of each oil series on time scale D4.

**Figure 6:**
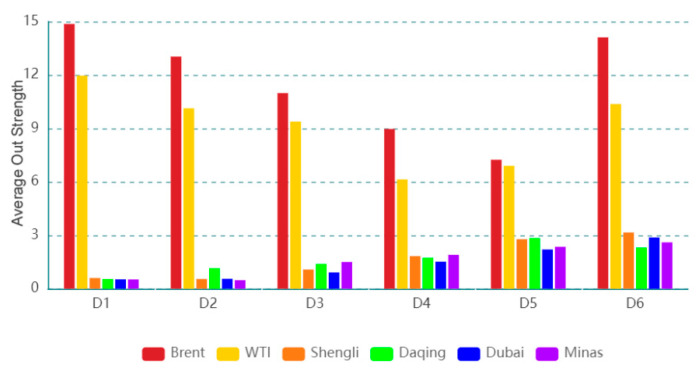
Average out strength of each oil series on different time scales.

The dynamic out strength for each oil series on a time scale (D4) is presented in Figure 5, and the dynamic out strength for the oil series on the other time scales is exhibited in the Appendix A. The results suggest that the importance of information spillover sent by the oil series to others changes with time. The dynamic features of the dynamic out strength at various time scales are different, indicating that the dynamic features are related to time scales. Figure 6 displays the average out strength of an oil series at different time scales. For instance, Brent has the greatest average out strength, and Minas has the smallest value on time scale D1. The result reflects that Brent, as a spillover transmitter, has the greatest importance of information spillover and Minas as a spillover transmitter has the smallest importance of information spillover. With the increase in the time scale, the average out strength of Brent (WTI) shows a downward trend, and that of Daqing (Shengli/Dubai/Minas) exhibits an upward trend. This indicates that the importance of Brent (WTI) as a spillover transmitter for information spillovers shows a downward trend and that of Daqing (Shengli/Dubai/Minas) as a spillover transmitter shows an upward trend with an increase in the time scale.

**Figure 7:**
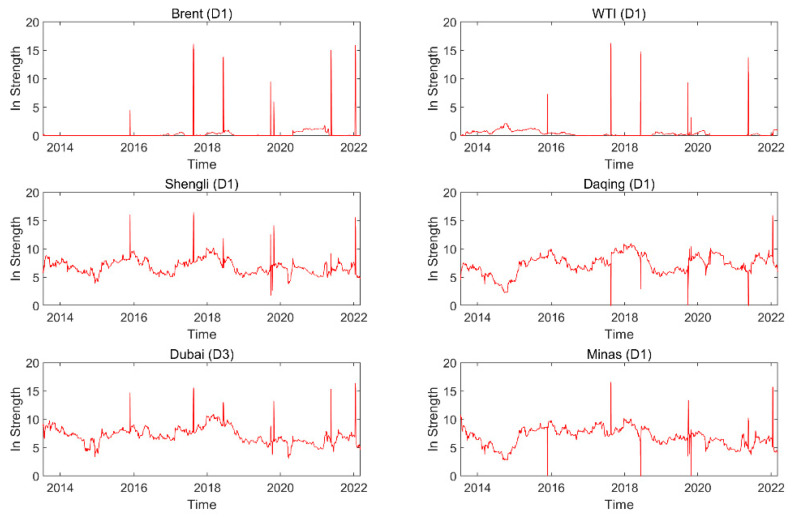
Dynamic in strength of each oil series on time scale D1.

**Figure 8:**
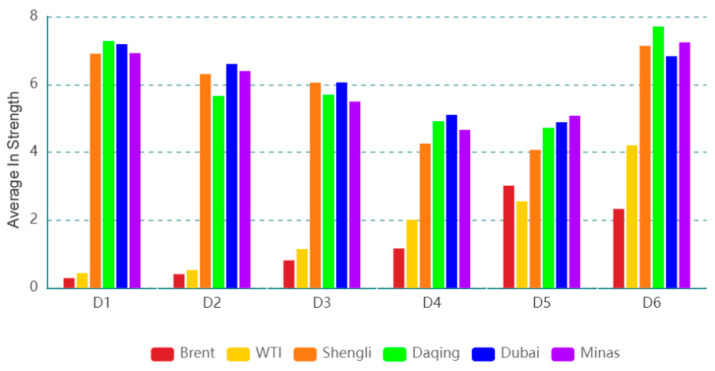
Average in strength of each oil series on different time scales.

Figure 7 shows the dynamic in strength for each oil series on a time scale (D1), and the dynamics in strength for the oil series on the other time scales are exhibited in the Appendix A. The results indicate that the importance of information spillover received by the oil series from other oil series is time-varying. The dynamic features of dynamic in strength at various time scales are different, indicating that the dynamic feature is related to time scales. Figure 8 presents the average in strength of an oil series at different time scales. For instance, Brent has the smallest average in strength, and Daqing has the greatest value on time scale D1. The result implies that Brent, as a spillover receiver, has the smallest importance of information spillover, and Daqing, as a spillover receiver, has the greatest importance of information spillover. With the increase in the time scale, the average in strength of Brent (WTI) shows an upward trend, and that of Shengli (Dubai) exhibits a downward trend. This indicates that the importance for information spillovers of Brent (WTI) as a spillover receiver shows an upward trend and that of Shengli (Dubai) as a spillover receiver shows a downward trend with the increase in the time scales.

### 3.2. Dynamic Structure of the Whole Oil System

**Figure 9:**
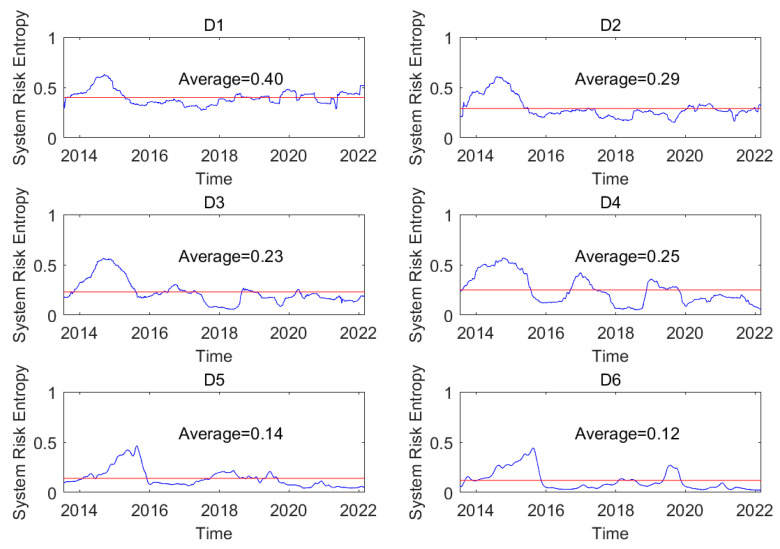
The dynamic system risk entropy in the whole system at different time scales.

This paper examines the total spillover and system risk entropy to measure the systematic risk of the whole oil system consisting of oil series when typical financial or economic events occur. For instance, the COVID-19 pandemic has affected global financial markets [38,39], especially WTI crude oil trades in negative for the first time. The total spillover indicates the degree of integration of the oil series, and system risk entropy implies the disorder of the oil series. Figure 9 shows the dynamic system risk entropy of a network at different time scales and indicates the time-varying systematic risk on the short-, medium-, and long-term time scales. For instance, the dynamic system risk entropy at time scale (D1) ranges from 0.28 to 0.63, where the mean of the system risk entropies of all the networks is 0.40. In addition, the mean value on the short-term time scale is greater than that on the long-term time scale. This indicates that the systematic risk based on entropy as a whole on the short-term time scale is smaller than that on the long-term time scale.

Furthermore, the trend of the system risk entropy during the whole sample period can be analyzed. For instance, the system risk entropy at time scale (D1) begins with the lower value and then spikes and declines. This result reflects that the indicator maintains a larger value compared with the mean value from the end of 2013 to the beginning of 2015, where the indicator decreases when the shale oil revolution in the United States in 2014 occurs. This suggests that the systematic risk based on entropy is lower during the period of global economic recovery and increases when crude oil prices plunge at the end of 2014. After that, the system risk entropy remains lower and exceeds the mean value until the end of 2019. The supply and demand in the global market change with time based on complex indicators, such as China–US trade frictions and the new agreement of OPEC, and the systematic risk was larger from 2015 to 2019. The system risk entropy became larger at the beginning of COVID-19 and then became smaller at the end of 2020 and again became larger at the second half of 2021. This result reflects that systematic risk based on entropy fluctuates over a large range during the period of the COVID-19 pandemic, indicating that it changes with higher uncertainty. The uncertain systematic risk entropy may give a sign of difficulty to hedge.

**Figure 10:**
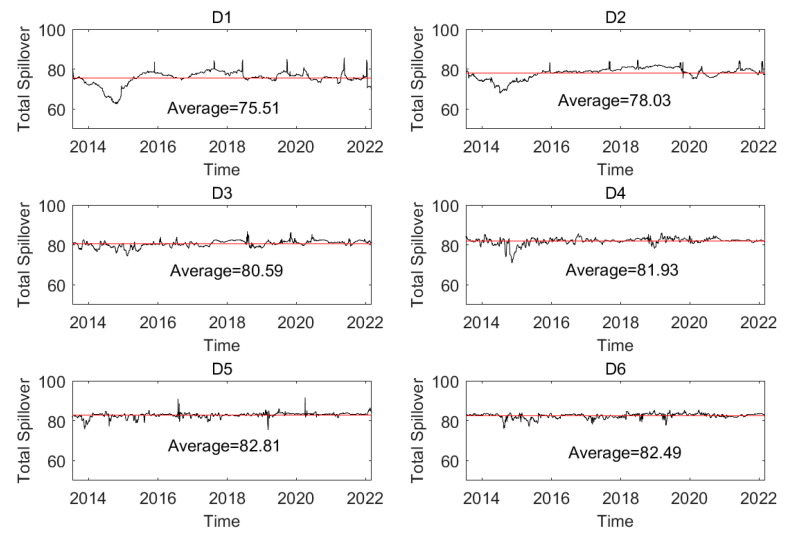
The dynamic total spillover of the whole system at different time scales.

The dynamic total spillover in a network at different time scales is given in Figure 10, and it suggests that the total spillover on the short-, medium-, and long-term time scales changes with time. For instance, the total spillover at time scale (D1) belongs to (62.47,85.62), and the mean value of the total spillovers of all the networks is 75.51. With the increase in time scale, the mean value increases, which implies that the integration of the oil series increases. This indicates that systematic risk based on total spillover in the short-term period is smaller than that on the long-term time scale.

The trend of the total spillover during the whole sample period can be analyzed. For instance, the total spillover at time scale (D1) begins with a greater value and then declines. It maintains a lower value from the end of 2013 to the second half of 2014, indicating that the integration of oil series is smaller, and the systematic risk based on total spillover is smaller. When the shale oil revolution in the United States in 2014 occurred, the total spillover became higher, indicating that the systematic risk was greater. After that, the total spillover maintained higher values until the second half of 2020. This indicates that the systematic risk based on total spillover was greater during that period from 2014 to the first half of 2020. From the beginning of the COVID-19 pandemic, the systematic risk based on total spillover fluctuated around the mean value in most sample time periods and sometimes had a larger or smaller value. This indicates that systematic risk based on the integration of the oil series maintains a mean value and sometimes is larger or smaller.

## 4. Conclusions and Discussions

This paper investigates the dynamic information spillover across international crude oil prices at different time scales based on a spillover network model combined with a wavelet transform and entropy theory. Specifically, a dynamic spillover network can be transformed from a multivariate oil series at a time scale, where the node is the oil price, and the weighted directed edge is the net information spillover from one price to the other. The proposed method cannot only detect the important role of an oil series at a scale but can also analyze the dynamic systematic risk from two perspectives at a time scale. The main conclusions can be described as follows:

First, the topological structure of a spillover network is measured to investigate the importance of a single oil series at a time scale. The dynamic structure of an oil series changes with time, suggesting that the importance of an oil price is time-varying. The importance of oil prices can be measured on a scale. The results show that WTI and Brent are important spillover transmitters, and others are important spillover receivers at all time scales, indicating that WTI and Brent play important global benchmark roles. With the increase in time scales, both the number of neighbors and the importance of spillovers of Brent and WTI as spillover transmitters show downward trends. The importance for information spillovers of China-Shengli and Dubai as spillover receivers also shows a downward trend. This paper provides new evidence for explaining the different roles of reference benchmark oil prices.

Second, systematic risk can be measured by analyzing dynamic system risk entropy and total spillover across oil series. The system risk entropy represents the disorder of the oil system consisting of oil series, and the total spillover represents the degree of integration of oil series. The results indicate that systematic risk at the short-term time scale is smaller than that at the long-term time scale. The fluctuation trends of these two indicators during different time scales can be found when the typical economy and financial events occur. For instance, at a time scale of 2–4 days, the system risk entropy fluctuates over a large range during COVID-19, and the degree of integration of the oil series maintains a mean level in most of that period.

The results provide important implications for policymakers and energy-related market investors at different time scales. They should pay attention to the differences in the spillover mechanism among oil series and the dynamic feature of systematic risk at short-, medium-, and long-term time scales. Market investors should efficiently hedge strategies to reduce investment risk, and policymakers should protect against the contagion effect due to the dynamic systematic risk.

This paper established the information spillover network based only on the DY model, wavelet transform, and entropy theory. Future research can use advanced entropy theories or establish other relationships across oil series based on econometric models to investigate the dynamic process of systematic risk.

## Figures and Tables

**Table 1:** The distribution characteristics of the crude oil prices.

Series	Max	Min	Mean	Standard Deviation
WTI	123.70	−37.63	64.56	21.73
Brent	127.98	19.33	70.53	24.34
Shengli	130.13	19.67	65.81	24.82
Daqing	124.19	12.35	64.58	25.42
Dubai	127.91	13.56	68.35	24.13
Minas	121.60	11.56	67.44	25.74

**Table 2:** The scales of the reconstructed oil time series.

Time Scales	Time–Frequency Domain	Term
D1	2–4 Days	Short Term
D2	4–8 Days	
D3	8–16 Days	Medium Term
D4	16–32 Days	
D5	32–64 Days	Long Term
D6	64–128 Days	
V	More than 128 Days	Trend Level

**Figure A1:**
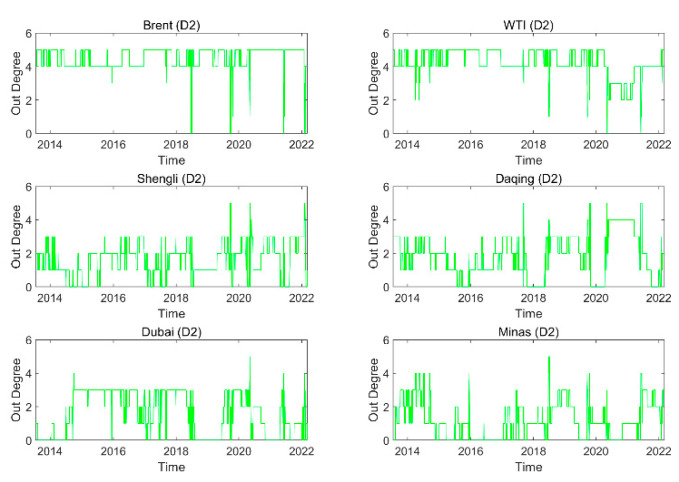
Dynamic out degree of each oil series on time scale D2.

**Figure A2:**
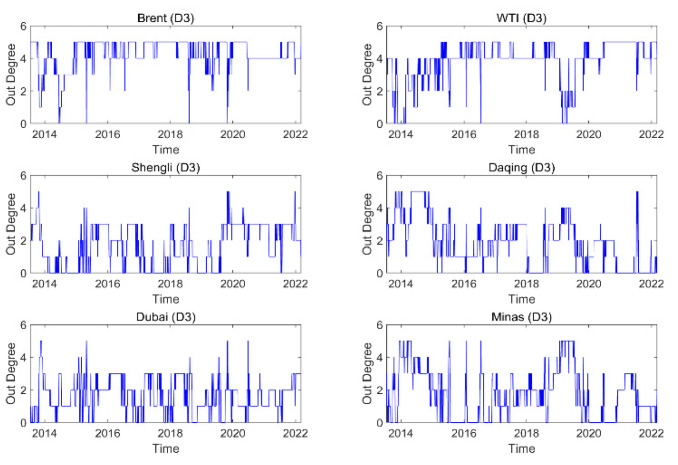
Dynamic out degree of each oil series on time scale D3.

**Figure A3:**
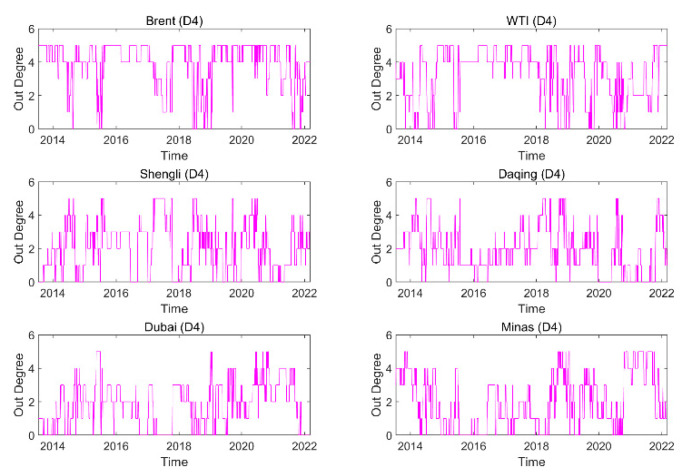
Dynamic out degree of each oil series on time scale D4.

**Figure A4:**
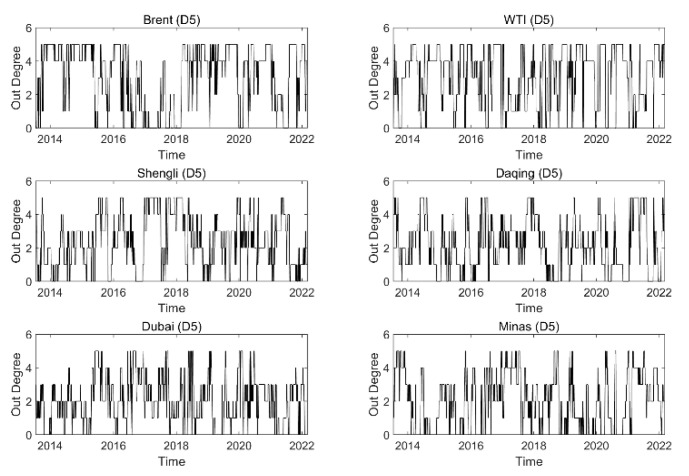
Dynamic out degree of each oil series on time scale D5.

**Figure A5:**
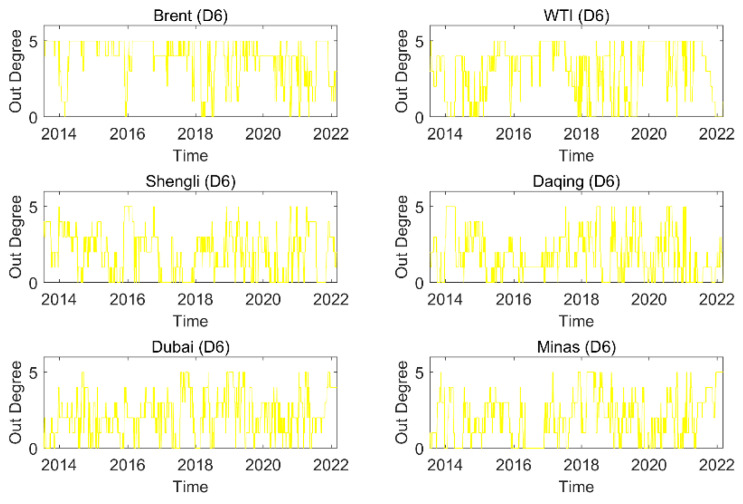
Dynamic out degree of each oil series on time scale D6.

**Figure A6:**
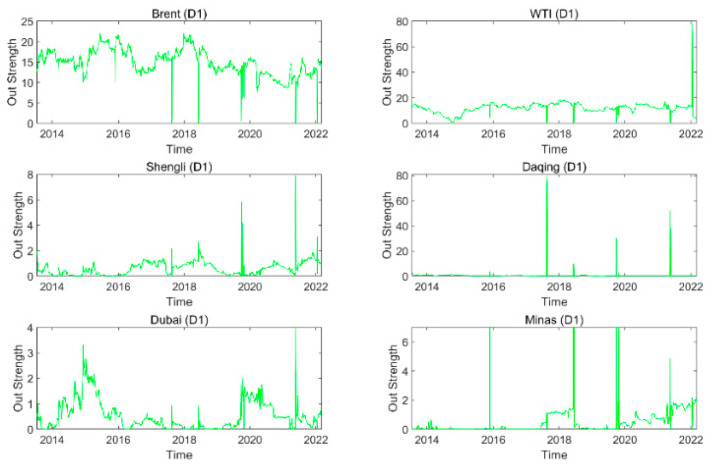
Dynamic out strength of each oil series on time scale D1.

**Figure A7:**
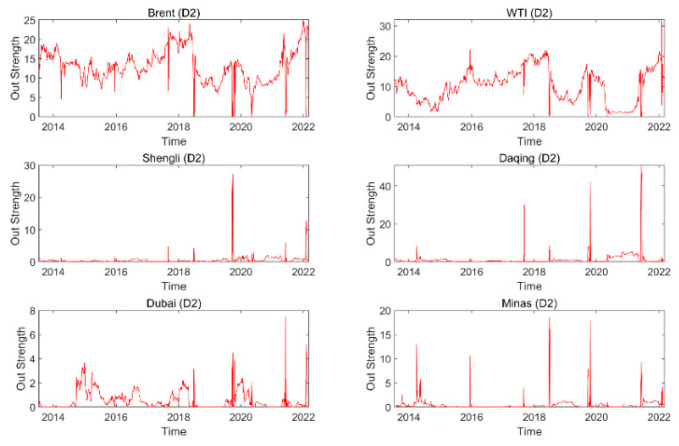
Dynamic out strength of each oil series on time scale D2.

**Figure A8:**
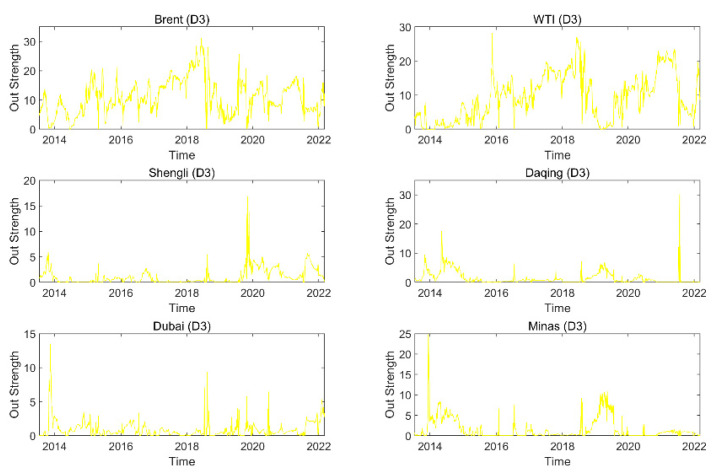
Dynamic out strength of each oil series on time scale D3.

**Figure A9:**
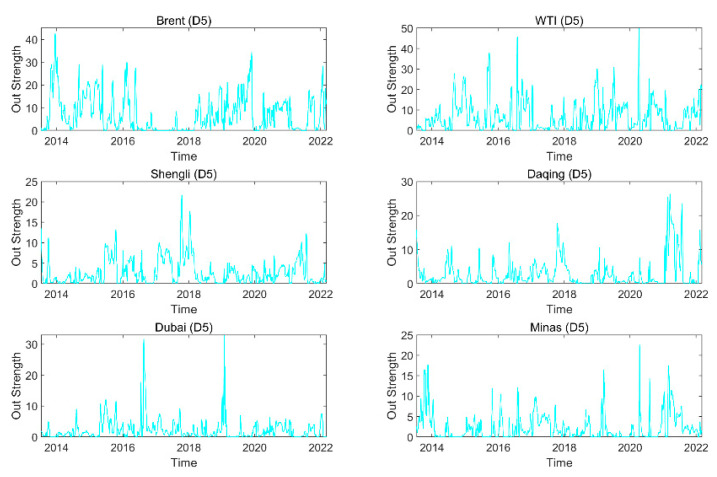
Dynamic out strength of each oil series on time scale D5.

**Figure A10:**
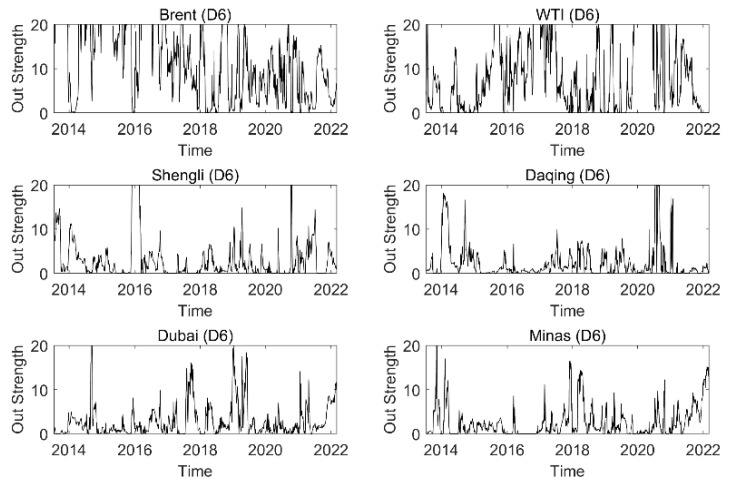
Dynamic out strength of each oil series on time scale D6.

**Figure A11:**
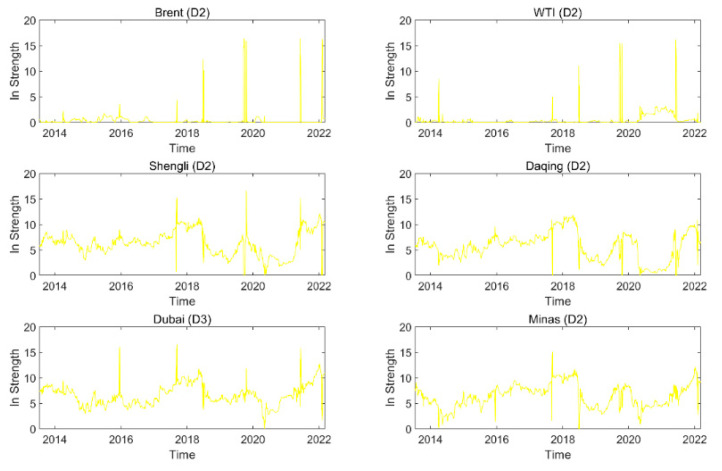
Dynamics in strength of each oil series on time scale D2.

**Figure A12:**
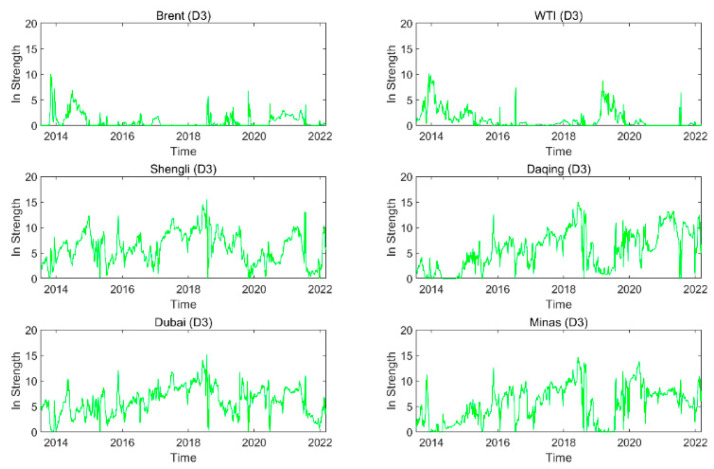
Dynamics in strength of each oil series on time scale D3.

**Figure A13:**
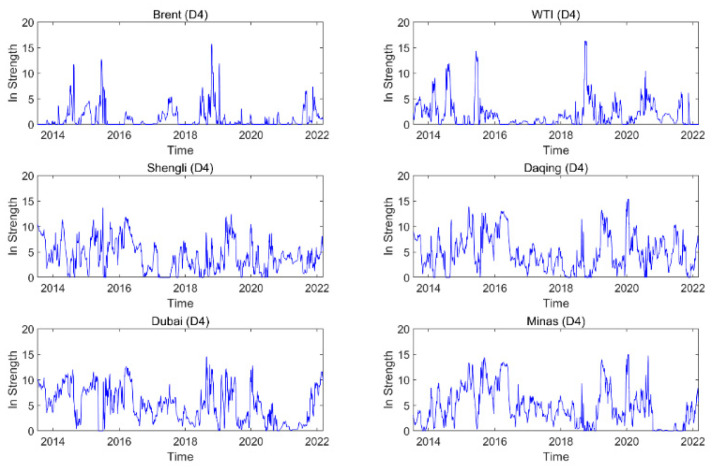
Dynamics in strength of each oil series on time scale D4.

**Figure A14:**
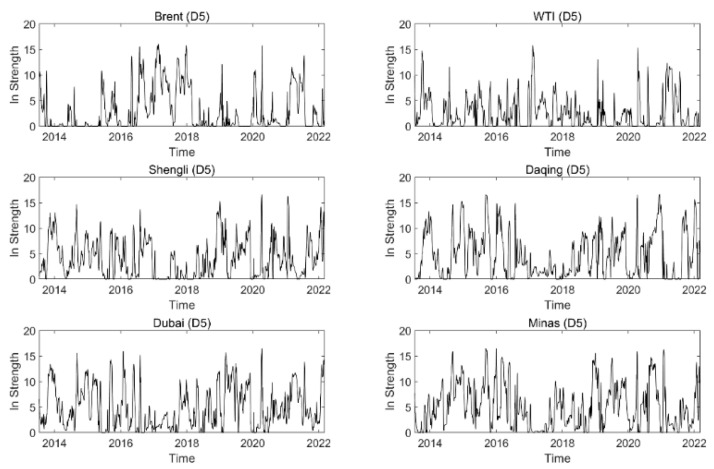
Dynamics in strength of each oil series on time scale D5.

**Figure A15:**
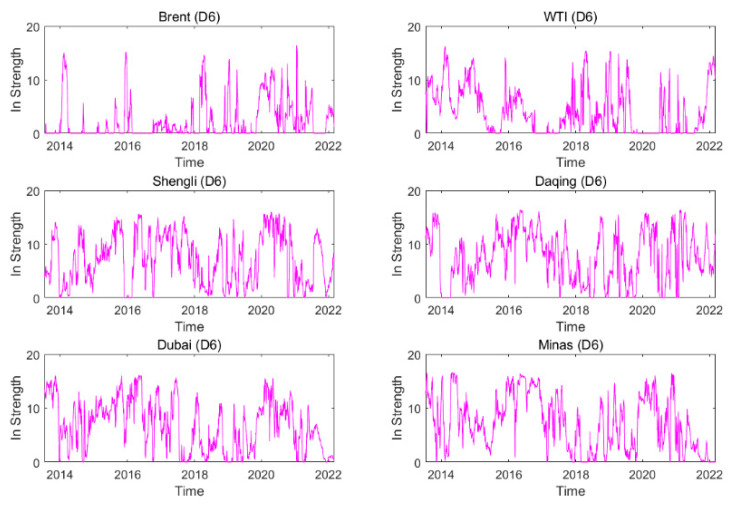
Dynamics in strength of each oil series on time scale D6.
